# An Exploration of Challenges in Routine Emergency Care With Delayed Presentations of Blunt Abdominal Trauma

**DOI:** 10.7759/cureus.92675

**Published:** 2025-09-18

**Authors:** Bilal Fattani, Rana Muhammad Umar Rizwan, Kashaf Ali

**Affiliations:** 1 Medicine, Jinnah Medical and Dental College, Karachi, PAK; 2 General Surgery, Jinnah Hospital, Lahore, Allama Iqbal Medical College, Lahore, PAK; 3 Pathology, Indus Medical College and Hospital, Tando Muhammad Khan, PAK

**Keywords:** abdominal injuries, emergency medical services, health services, time-to-treatment, treatment outcome

## Abstract

Background: Delayed presentation among patients with blunt abdominal trauma (BAT) constitutes one of the biggest challenges in the emergency care field, particularly in low-resource regions. Delays may increase the risk of developing intra-abdominal infections, increase the need for surgical procedures, and deteriorate the overall outcomes. The purpose of this study was to determine the prevalence of delayed presentations, major predisposing factors, and their contribution to complications in BAT patients.

Methods: It was a prospective observational study conducted over six months using a sample of 120 patients who presented to the emergency department with a BAT. Patients were divided into two groups based on time for presentation: early (less than six hours of injury, n = 83) and delayed presenters (greater than six hours, n = 37). Information regarding demographics, mechanism of injury, clinical signs, diagnostics, treatment modalities, complications, and outcomes was gathered. The effects of delay on morbidity and mortality were evaluated. Utilizing Statistical Package for the Social Sciences version 26.0 (IBM Corp., Armonk, NY), the chi-square tests were performed on the categorical variables and independent t-tests on the continuous variables. A p value of <0.05 was used for significance.

Results: Around one-third of the patients presented late. The most important contributing factors included lack of awareness, 16 (43.2%), transportation issues, 10 (27.0%), and first treatment with nonspecialist providers, seven (18.9%), all of which were indicative of long delays (p < 0.05). The delayed presenters were associated with increased intra-abdominal infections, nine (24.3%) vs. seven (8.4%), p = 0.011, and surgical intervention, 22 (59.5%, confidence interval, CI: 43.5-73.7) vs. 31 (37.3%, CI: 27.7-48.1), p = 0.021.

Conclusion: The risk of complications and operative requirements in patients with BAT is largely determined by time to hospital presentation. To minimize unnecessary delays, community education, emergency transportation, and simplified referral are often essential.

## Introduction

Blunt abdominal trauma (BAT) continues to present morbidity and mortality, particularly in developing countries, where road traffic accidents rank highest among these injuries [1]. Although post-traumatic care and prehospital services have improved, delayed presentations (greater than six hours after injury) still present major clinical burdens on emergency departments [2]. The time to definitive treatment is prolonged due to various factors that have been shown to increase the risk of complications, including intra-abdominal infections and emergency surgical procedures [3]. This is due to a lack of public awareness, insufficient infrastructure, and the use of nonspecialist providers [4]. According to multicenter audits, 35% of patients with BAT arrive after the critical hour (the immediate period after injury when timely medical intervention is most likely to improve outcomes), and this number was directly linked to admissions to the ICU and long hospital stays [5]. Despite enhanced outcomes with early diagnosis through rapid imaging in high-resource countries, these approaches have proven to be ineffective when patients present late [6].

Available evidence indicated that successful community education interventions, reinforcement of prehospital responses, and maximizing hospital preparedness require understanding local patterns of delayed presentation and their predictive factors [7]. Despite the advances in the development of trauma networks in several countries, the recent high prevalence of late presenters highlights the importance of evidence-informed interventions that address the unique settings [8].

The purpose of this study was to identify the prevalence of late presentations among patients with BAT. This study also aimed to assess the factors associated with delayed presentations, along with the relationship between presentation time and clinical outcomes, complications, surgical intervention, and mortality.

## Materials and methods

The frequency, causes, and clinical consequences of delayed presentation in patients with BAT were evaluated in this prospective observational study, which was conducted in the emergency department and trauma unit of Ziauddin University, Karachi, over six months from October 2023 to March 2024 after ethical approval (ref: 4442MS/21). All participants or their legal representatives provided informed consent.

A consecutive nonprobability sampling method was employed to recruit all the eligible patients who presented with BAT during the study period. The sample size of 120 was estimated using OpenEpi version 3.0.0 (released, 2013, Atlanta, GA; Dean AG, Sullivan KM, Soe MM. OpenEpi: Open Source Epidemiologic Statistics for Public Health. www.OpenEpi.com, updated 2013/04/06) based on the anticipated prevalence of delayed presentation of 30%-35%, at a 95% confidence level, and a margin of error of 8%; the minimum required sample size was 120 patients [9]. The inclusion criteria of patients included patients aged 18 years and older, who presented with confirmed BAT due to road traffic accidents, falls, and direct blunt force. The exclusion criteria were penetrating abdominal trauma, isolated limb or head injury without abdominal involvement, prior history of intra-abdominal surgical disease, absent data, or death, and discharge against medical advice before proper evaluation.

The participants were divided into two groups: early presenters (less than six hours of injury) and delayed presenters (greater than six hours). The chosen six-hour threshold represents previous trauma literature findings showing that delays in this range correlate with higher complications and poorer patient outcomes [10]. The time from injury to hospital arrival was confirmed through patient testimonies, referrals, and records to maintain compliance. Demographics, mechanism of injury, clinical features, imaging, treatment, and outcomes were documented. Conventional diagnostic methods, including abdominal ultrasound and contrast-enhanced CT, were performed according to institutional procedures.

The data were analyzed by Statistical Package for the Social Sciences version 26.0 (released 2019, IBM Corp., Armonk, NY). Chi-square tests were applied to categorical variables and independent t-tests to continuous variables to assess relationships between presentation time and outcomes. The statistical significance was considered at a p value of <0.05.

## Results

The most common reasons associated with delayed presentation were insufficient awareness, transport challenges, and initial care by nonspecialists. The study involved 120 patients who had BAT. Patients were divided into two groups: early (within six hours) and late (more than six hours) presenters. There was no significant difference in baseline demographic data between the two groups in terms of age, gender, and mechanism of injury. Demographic characteristics of study participants are given in Table 1.

**Table 1:** Demographic characteristics of study participants SD: standard deviation

Parameter	Early presenters (n = 83)	Delayed presenters (n = 37)	Test used	Test value	p value
Mean age (years), mean ± SD	35.4 ± 12.6	37.1 ± 11.9	Independent t-test	t = 0.70	0.486
Male, n (%)	64 (77.1%)	30 (81.1%)	Chi-square test	χ² = 0.27	0.605
Road traffic accident, n (%)	50 (60.2%)	21 (56.8%)	Chi-square test	χ² = 0.12	0.729

Baseline demographics and characteristics of injuries were relatively similar in early and delayed presenters. Mean age values were 35.4 ± 12.6 years (early presenters) and 37.1 ± 11.9 years (delayed presenters), with no statistical difference (p = 0.486). The difference in the proportion of male patients was not significant, 64 (77.1%) in early vs. 30 (81.1%) in delayed presenters (p = 0.605). The most common mechanism of injury was road traffic accidents in both early and delayed presenters, 50 (60.2%) and 21 (56.8%), respectively (p = 0.729). Reasons for delay among delayed presenters are indicated in Table 2.

**Table 2:** Reasons for delay among delayed presenters (n = 37) ^*^Statistical significance at <0.05

Lack of awareness	Transportation difficulty	Initial nonspecialist treatment	Test used	Test value	Significance (p value)
16 (43.2%)	10 (27.0%)	7 (18.9%)	Chi-square	8.50	0.036^*^

Among 37 delayed presenters, the most common reason for late arrival was a lack of awareness, 16 (43.2%). It was followed by transportation difficulties, 10 (27.0%), and nonspecialist treatment, seven (18.9%). These were major modifiable factors based on the statistically significant p value (p = 0.036). Table 3 illustrates the time for presentation of study participants and associated factors.

**Table 3:** Time for presentation and associated factors (n = 37) ^*^Statistical significance at <0.05 SD: standard deviation

Factor	Mean delay (hours), mean ± SD	Test used	Test value (t)	p value
Transportation delay	8.5 ± 3.2	Independent t-test	5.04	<0.001^*^
Awareness-related delay	9.1 ± 2.8	Independent t-test	4.67	<0.001^*^
Initial nonspecialist treatment delay	7.8 ± 3.0	Independent t-test	3.85	0.001^*^

The average delay was 9.1 ± 2.8 hours for patients with a lack of awareness, 8.5 ± 3.2 hours among patients with transportation barriers, and 7.8 ± 3.0 hours among patients who had nonspecialist care. All these causes of delays were statistically significant (p < 0.001). These results highlight the importance of implementing measures to enhance timely access to affordable emergency transport and community education. Clinical outcomes and complications are presented in Table 4.

**Table 4:** Clinical outcomes and complications ^*^Statistical significance at <0.05 CI: confidence interval

Outcome	Early presenters (n = 83)	Delayed presenters (n = 37)	Test used	χ² value	Significance (p value)
Intra-abdominal infection	7 (8.4%); CI: 4.1-16.4	9 (24.3%); CI: 13.4-40.1	Chi-square test	6.42	0.011^*^
Need for surgical intervention	31 (37.3%); CI: 27.7-48.1	22 (59.5%); CI: 43.5-73.7	Chi-square test	5.30	0.021^*^
ICU admission	10 (12%); CI: 6.7-20.80	7 (18.9%); CI: 9.5-34.2	Chi-square test	1.09	0.296
Mortality	2 (2.4%); CI: 0.7-8.4	4 (10.8%); CI: 4.3-24.7	Chi-square test	2.76	0.097

The delayed group had a higher rate of ICU admission, 7 (18.9%, confidence interval, CI: 9.5-34.2) vs. 10 (12.0%, CI: 6.7-20.80), p = 0.296, and mortality, 4 (10.8%, CI: 4.3-24.7) vs. 2 (2.4%, CI: 0.7-8.4), p = 0.097, but these were not statistically significant. These findings suggest that delay primarily contributes to an elevated risk of infection and surgical intervention.

## Discussion

The purpose of this study was to determine the frequency, contributory factors, and clinical implications of late presentations in patients with BAT and to establish areas within which emergency care delivery can be effectively enhanced. These results align with the study, which demonstrated the limited knowledge about internal injuries among people playing a significant role in delays in low-resource settings [11]. The relationship between transportation challenges and long delays aligns with the findings of another study, which revealed that low-quality emergency transportation was one of the key factors in delivering trauma care in rural regions [12].

This study also found that more patients received late treatment under the care of nonexperts, which also reflects the results from the previous literature, which emphasized that patients initially managed improperly may not show abdominal injury progression [13]. Moreover, significant intra-abdominal infections and surgical requirements in delayed presenters were observed. This is consistent with a study that discovered increased complications and resource requirements in the late diagnosis of hollow viscus injuries [14]. Although the number of ICU admissions and mortality was higher in the delayed group, it was not statistically significant. These findings are consistent with observations that delayed presentation alone may not be entirely responsible for mortality [15].

These findings also support the idea presented in another study that referral practices can help reduce inappropriate initial treatment [16]. A study indicated that strong community education can contribute to a reduction in delays, which is inconsistent with the results of this study, which demonstrated a lack of awareness, even with local awareness campaigns, suggesting interventions may require specific community considerations [17]. Another study also showed that the burden of delays caused by transport issues can be alleviated with well-coordinated trauma networks, indicating the necessity of better emergency systems [18].

The limitations of this study include its single-center design and small sample size, which affect the generalizability of the results and introduce selection bias. Dependence on patients to recall the time of injury can result in recall bias. Moreover, trauma severity scales were not utilized, which reduces valid comparisons between the groups. There were unmeasured confounding factors, including socioeconomic status, comorbidities, and variability in prehospital care, that could have affected both the presentation time and the outcome. Furthermore, no data on long-term outcomes were assessed; only the short-term, inhospital outcomes were evaluated. It is proposed that future multicenter studies with standardized severity scoring, larger sample, and extended follow-up be conducted to validate these findings.

## Conclusions

This study has shown that delay in the hospital presentation of patients with BAT to the emergency department is common and significantly associated with the high risks of intra-abdominal infections and the need for surgical intervention. The main causes of the delay were identified as the lack of community awareness, transport issues, and the initial treatment by nonspecialist care providers who may fail to identify hidden injuries, representing modifiable targets for intervention.

Addressing these factors with public education, investment in emergency transport infrastructure, and structured referral pathways may substantially improve outcomes in similar resource-limited settings. Although the differences in ICU admission and mortality were not statistically significant, the trend supports the idea that larger sample sizes may reveal more pervasive outcomes of delayed care. Future multicenter studies should be more focused on context-specific strategies, integrate trauma severity scores, and adjust for confounding factors to facilitate earlier identification and treatment of blunt abdominal injuries.
